# Complete Genome Sequences of *Vibrio cholerae*-Specific Bacteriophages 24 and X29

**DOI:** 10.1128/genomeA.01013-17

**Published:** 2017-11-16

**Authors:** Sudhakar G. Bhandare, Andrew Warry, Richard D. Emes, Steven P. T. Hooton, Paul A. Barrow, Robert J. Atterbury

**Affiliations:** a University of Nottingham, School of Veterinary Medicine and Science, Sutton Bonington, United Kingdom; b University of Nottingham, Advanced Data Analysis Centre, Sutton Bonington, United Kingdom; c University of Nottingham, School of Biosciences, Division of Food Science, Sutton Bonington, United Kingdom

## Abstract

The complete genomes of two *Vibrio cholerae* bacteriophages of potential interest for cholera bacteriophage (phage) therapy were sequenced and annotated. The genome size of phage 24 is 44,395 bp encoding 71 putative proteins, and that of phage X29 is 41,569 bp encoding 68 putative proteins.

## GENOME ANNOUNCEMENT

Genome sequencing of bacteriophage therapy candidates is required to determine whether they carry genes related to virulence, antimicrobial resistance, and/or lysogeny. Two phages which infect *Vibrio cholerae* (24 and X29) were obtained for sequencing from the Felix d’Herelle Reference Centre for Bacterial Viruses, Canada, prior to initiating phage therapy studies. Phage 24 was isolated from sewage in Bangladesh (1), while phage X29 was isolated from cholera stools in India (2). Both of these phages belong to the *Myoviridae* family. The measurements of head diameter and tail length for these phages are 64 nm and 79 nm (phage 24) and 64 nm and 142 nm (phage X29), respectively (3).

The genomic DNA of these phages was extracted using the Promega DNA extraction kit (A7280 Wizard DNA cleanup system) (4). The DNA samples were prepared according to the Nextera DNA sample preparation guide and sequenced on an Illumina MiSeq platform (2 × 250-bp paired-end reads with 120× coverage). Complete genome sequences were assembled using SPAdes version 3.1.0 (5). Genome annotation was performed using the Rapid Annotations using Subsystems Technology (RAST) server (6) with some additional manual curation. The translated sequence of each predicted gene was compared to known proteins using BLASTp analysis (7), and conserved protein motifs were identified using the Pfam database (8). The genomes were scanned for tRNAs using tRNAscan-SE (9) and ARAGORN (10). The phage 24 genome was found to be circularly permuted with terminal redundancy, in contrast to phage X29, which has defined ends indicated by steep drop-offs in read coverage at the contig ends.

The genome size of phage 24 is 44,395 bp with a GC content of 45.4%. It contains 71 predicted coding DNA sequences (CDSs), of which 19 could be assigned putative functions. Phage 24 has 99% identity with *Vibrio* phage CP-T1 (11), the major difference being an in-frame 75-bp deletion in the pentapeptide repeat-containing protein annotated in phage 24. The conserved proteins identified can be grouped as structural head proteins, packaging proteins, phage tail proteins, and DNA replication proteins. The head proteins were a putative head protein, a major capsid protein, and a phage portal protein. Packaging proteins were small- and large-terminase subunits. The phage tail proteins were a tail fiber/lysozyme protein and a baseplate protein. The DNA replication proteins were a helicase, a primase, and a DNA polymerase.

Phage X29 has a genome size of 41,569 bp with a GC content of 46% containing 68 CDSs; predicted functions could be assigned to 36 of them. Phage X29 shares 99% identity with *Vibrio* phage Phi 2 (KJ545483). It contains a conserved translational frameshift in the coding sequences located upstream of its tape measure protein that are analogous to the lambda proteins gpG and gpGT (12). The genome codes for phage structural proteins and several DNA-binding proteins with helix-turn-helix domains. The tail fiber and shaft proteins were a tail length tape measure protein, a putative phage tail protein, a tail fiber assembly-like protein, and a tail fiber protein. The coat proteins were a major capsid protein, a head maturation protease, and a portal protein. Lysogeny-related proteins were phage integrases and a Cro protein homolog. Analysis of both phage genomes revealed an absence of tRNAs.

### Accession number(s).

This whole-genome shotgun project has been deposited at GenBank under the accession no. KJ572844 (phage 24) and KJ572845 (phage X29). The versions described in this paper are the second versions, KJ572844.2 and KJ572845.2, respectively.
